# Endothelial Protein C Receptor Gene Variants Not Associated with Severe Malaria in Ghanaian Children

**DOI:** 10.1371/journal.pone.0115770

**Published:** 2014-12-26

**Authors:** Kathrin Schuldt, Christa Ehmen, Jennifer Evans, Juergen May, Daniel Ansong, Juergen Sievertsen, Birgit Muntau, Gerd Ruge, Tsiri Agbenyega, Rolf D. Horstmann

**Affiliations:** 1 Department of Molecular Medicine, Bernhard Nocht Institute for Tropical Medicine, Hamburg, Germany; 2 Infectious Disease Epidemiology Group, Bernhard Nocht Institute for Tropical Medicine, Hamburg, Germany; 3 School of Medical Sciences, Kwame Nkrumah University of Science and Technology, Kumasi, Ghana; Barcelona Centre for International Health Research/Hospital Clinic/IDIBAPS/University of Barcelona, Spain

## Abstract

**Background:**

Two recent reports have identified the Endothelial Protein C Receptor (EPCR) as a key molecule implicated in severe malaria pathology. First, it was shown that EPCR in the human microvasculature mediates sequestration of *Plasmodium falciparum*-infected erythrocytes. Second, microvascular thrombosis, one of the major processes causing cerebral malaria, was linked to a reduction in EPCR expression in cerebral endothelial layers. It was speculated that genetic variation affecting EPCR functionality could influence susceptibility to severe malaria phenotypes, rendering *PROCR*, the gene encoding EPCR, a promising candidate for an association study.

**Methods:**

Here, we performed an association study including high-resolution variant discovery of rare and frequent genetic variants in the *PROCR* gene. The study group, which previously has proven to be a valuable tool for studying the genetics of malaria, comprised 1,905 severe malaria cases aged 1–156 months and 1,866 apparently healthy children aged 2–161 months from the Ashanti Region in Ghana, West Africa, where malaria is highly endemic. Association of genetic variation with severe malaria phenotypes was examined on the basis of single variants, reconstructed haplotypes, and rare variant analyses.

**Results:**

A total of 41 genetic variants were detected in regulatory and coding regions of *PROCR*, 17 of which were previously unknown genetic variants. In association tests, none of the single variants, haplotypes or rare variants showed evidence for an association with severe malaria, cerebral malaria, or severe malaria anemia.

**Conclusion:**

Here we present the first analysis of genetic variation in the *PROCR* gene in the context of severe malaria in African subjects and show that genetic variation in the *PROCR* gene in our study population does not influence susceptibility to major severe malaria phenotypes.

## Introduction

Endothelial protein C receptor (EPCR) is found at the surface of the endothelial cells of diverse tissue origin [1]. It functions as the principal regulatory molecule for protein C that as activated protein C (APC) exerts anticoagulant and cytoprotective functions, thereby maintaining the integrity of endothelia.

Recently, two independent studies provided evidence for an implication of EPCR in severe malaria (SM) and cerebral malaria (CM) pathologies [2], [3]. With regard to SM, EPCR was identified as an endothelial receptor for certain binding cassettes of *Plasmodium falciparum* erythrocyte membrane protein 1 (PfEMP1) [2]. Turner and colleagues pinpointed the binding site of PfEMP1, which appears to be located near or directly at the domain mediating EPCR binding to protein C. It was postulated that, by occupying the protein C binding site, EPCR-mediated parasite adhesion could impair the cytoprotective and anti-inflammatory pathways. In consequence, a disruption of the endothelial layer could evoke vascular leakage, and, when these processes occur in cerebral microvessels, may result in brain hemorrhages typically observed in CM pathology. Further, expression levels of the transcript encoding the EPCR binding domain of PfEMP1 were shown to be significantly higher in *P. falciparum* isolates from children with CM or severe malaria anemia (SMA) compared to children with uncomplicated malaria [4]. Hence, there is additional indirect evidence for a link between PfEMP1 binding to EPCR and CM and SMA pathologies.

The second more recent study reported on a malaria-induced reduction of EPCR expression at the surface of cerebral endothelial layers in autopsies from Malawian children affected by CM [3]. Additionally, a low constitutive expression of EPCR was observed particularly in the brain. These findings might serve as an explanation for the organ-specific pathology in CM, which is induced by cytoadherence of *Plasmodium*-infected red blood cells (iRBCs) in brain microvessels to a wide range of additional receptors [5]. It was suggested that a low capacity of EPCR to activate protein C caused by adherence of iRBCs and adjacent cleavage of EPCR could provoke a proinflammatory and procoagulant state in affected tissues.

*PROCR*, the gene encoding EPCR, is located on chromosome 20 and spans approximately 6 kb of genomic DNA [6]. The gene comprises four exons. Exon I encodes an untranslated 5′ region (5′-UTR) and a signal peptide, exons II and III the extracellular domain of EPCR, and exon IV the transmembrane domain, the cytoplasmic tail and the 3′ untranslated region (3′-UTR).

Until today human genetic variation in *PROCR* has primarily been assessed in the context of common thrombotic disorders, such as cardiovascular disease and venous thrombosis [7], [8]. For the most part these studies examined a functionally relevant single nucleotide polymorphism (SNP) in *PROCR*, variant rs867186 A>G, which is located in exon IV. This SNP causes a serine-glycine substitution (S219G), and the G allele was associated with increased plasma levels of soluble EPCR (sEPCR), Factor VII, and protein C [9]–[11]. The G allele of rs867186 distinctly tags the *PROCR* haplotype A3. This haplotype was also associated with elevated plasma levels of sEPCR [12] and high levels of protein C [13]. A second functional haplotype, *PROCR* H1, was associated with higher amounts of APC in plasma and was found to be protective against venous thromboembolism [14]. In contrast, the A3 haplotype has been identified as a genetic risk factor for venous thrombosis, and it was hypothesized that a balanced polymorphism in *PROCR* could confer protection against SM at the cost of a higher risk of thrombotic disease [2]. Moreover, in a recent paper Aird *et al*. speculated that the same functional haplotype could also have a specific protective effect against CM, because an increased level of sEPCR could impair cytoadhesion of infected red blood cells to cerebral endothelial cells and hence prevent severe pathology in the brain [15].

Taken together, there is substantial evidence that human genetic variation affecting EPCR functionality could influence severe malaria phenotypes, rendering *PROCR* a promising candidate for an association study. Here, we assessed the influence of common, rare, and haplotypic genetic variation in the human *PROCR* gene on SM phenotypes in a large case-control study comprising more than 3,700 subjects [16] from the Ashanti Region in Ghana, West Africa.

## Results

Variant discovery in the regulatory and coding regions of the *PROCR* gene was conducted in 3,771 unrelated individuals from the Ashanti Region in Ghana. The study group included 1,905 children with SM, 431 of whom were classified as CM cases, 1,226 as SMA cases, and 1,866 apparently healthy control individuals. As a result of a high resolution melting (HRM) screen, a total of 41 genetic variants were detected, of which 17 were novel. Fifteen variants were found to be singletons, 17 had a minor allele frequency (MAF) below 1% and nine variants were found to have a MAF of 1% or greater (Table 1). Six SNPs in exonic regions caused non-synonymous amino acid exchanges in the receptor protein. One of these, a substitution of an aspartic acid by a glycine residue at position 23 (D23G) was previously unknown.

**Table 1:** Genetic variants in the *PROCR* gene found in 3,771 Ghanaian children.

	SNP ID	Position Chr19 (build37)	Major/Minor Allele	Region	Relative position^a^	AA exchange	Novel	MAF cases (N = 1,905)	MAF controls (N = 1,866)
1	ss1457457875	33758811	G/A	5′-UTR	c.-1147		YES	-	Singleton
2	rs113601425	33758824	C/T		c.-1134			0.007	0.007
3	ss1457457916	33758834	A/G		c.-1124		YES	0.001	0.001
4	ss1457457937	33758983	C/G		c.-975		YES	0.001	0.002
5	rs112681065	33759055	C/G		c.-903			0.006	0.008
6	ss1457457959	33759070	TA/-		c.-888		YES	Singleton	-
7	ss1457457984	33759072	CACA/-		c.-886		YES	Singleton	-
8	ss1457458010	33759093	C/A		c.-865		YES	0.002	0.001
9	rs115088244	33759104	G/T		c.-854			0.077	0.084
10	rs146473859	33759128	C/G		c.-830			0.001	0.001
11	ss1457458032	33759145	T/C		c.-813		YES	-	Singleton
12	rs2069940	33759272	C/G		c.-686			0.018	0.018
13	rs2069941	33759386	C/T		c.-572			0.013	0.012
14	ss1457458053	33759599	A/G		c.-359		YES	Singleton	-
15	rs113347910	33759639	C/A		c.-319			0.038	0.028
16	rs372603647	33759656	C/-		c.-301			-	Singleton
17	rs139617753	33759867	C/G	Ex1-UTR	c.-91			-	Singleton
18	ss1457458073	33759903	C/T		c.-55		YES	0.001	0.001
19	ss1457458095	33759972	G/A	Ex1	c.15	L5L	YES	0.001	0.001
20	rs145917629	33759978	G/A		c.21	P7P		0.004	0.003
21	ss1457458118	33760025	A/G		c.68	D23G	YES	Singleton	-
22	rs2069948	33762489	T/C	In1	c.71-16			0.125	0.126
23	rs141487483	33762664	A/G	Ex2	c.230	E77G		0.002	0.003
24	rs2069952	33763951	T/C	In2	c.323-20			0.132	0.14
25	COSM1222127	33764042	G/A	Ex3	c.395	E132K		-	Singleton
26	rs144485700	33764082	C/T		c.434	P145L		0.003	0.001
27	rs201040729	33764137	C/T		c.489	F163F		Singleton	-
28	rs116737636	33764292	C/T	In3	c.601+43			Singleton	-
29	rs148966205	33764458	T/A		c.602-43			0.001	-
30	rs145801152	33764542	G/A	Ex4	c.643	V215I		0.001	-
31	rs867186	33764554	A/G		c.655	S219G		0.057	0.059
32	rs9574	33764632	G/C	3'-UTR	c.*16			0.132	0.139
33	rs141265473	33764667	G/C		c.*51			0.006	0.004
34	ss1457458141	33764697	C/G		c.*81		YES	Singleton	-
35	ss1457458164	33764702	A/G		c.*86		YES	-	Singleton
36	ss1457458186	33764703	C/A		c.*87		YES	-	Singleton
37	rs146262354	33764758	C/G		c.*142			0.006	0.004
38	rs115542162	33764978	C/T		c.*362			0.032	0.030
39	ss1457458207	33765308	A/G		c.*692		YES	-	0.002
40	ss1457458230	33765332	A/G		c.*710		YES	-	Singleton
41	ss1457458251	33765355	A/G		c.*739		YES	0.001	0.001

AA, amino acid; Ex, exon; In, intron; MAF, minor allele frequency.

a Reference sequence NCBI NM_006404.4.

Association testing of the nine SNPs with MAFs ≥1% did not show any evidence for an association with SM, CM, or SMA (Table 2). A trend for an association with both, SM and SMA, was found for the A allele of promoter variant rs113347910 (SM: odds ratio [OR] 1.32, 95% confidence interval [CI] 1.01–1.73, p = 0.04; SMA: OR 1.39, 95% CI 1.01–1.90, p = 0.04), however, the associations did not hold after adjusting for multiple testing (p_corrected_ = 0.28, factor 7).

**Table 2:** Association tests of *PROCR* SNPs (MAF ≥1%) with severe malaria, cerebral malaria, and severe malaria anemia.

					Severe Malaria		Cerebral Malaria		Severe Malaria Anemia	
					N_cases_ = 1,905		N_cases_ = 431		N_cases_ = 1,226	
					N_controls_ = 1,866		N_controls_ = 1,866		N_controls_ = 1,866	
No.	SNP ID	Major/Minor Allele	HWE	MAF (aff/unaff)	OR (95% CI)^a^	p-value^a^	OR (95% CI)^a^	p-value^a^	OR (95% CI)^a^	p-value^a^
1	rs115088244	G/T	0.288	0.08/0.08	0.90 (0.75–1.07)	0.228	1.08 (0.83–1.41)	0.566	0.86 (0.69–1.05)	0.140
2	rs2069940	C/G	1	0.02/0.02	0.93 (0.65–1.35)	0.717	0.99 (0.59–1.74)	0.975	0.91 (0.60–1.40)	0.673
3	rs2069941	C/T	1	0.01/0.01	1.07 (0.73–1.61)	0.772	0.58 (0.25–1.38)	0.222	1.09 (0.37–1.80)	0.222
4	rs113347910	C/A	1	0.04/0.03	1.32 (1.01–1.73)	0.044	1.41 (0.94–2.11)	0.099	1.39 (1.01–1.90)	0.042
5	rs2069948	T/C	0.914	0.13/0.13	0.99 (0.86–1.15)	0.943	1.11 (0.89–1.38)	0.369	1.03 (0.87–1.22)	0.766
6	rs2069952	T/C	0.628	0.13/0.14	0.93 (0.81–1.07)	0.335	1.02 (0.82–1.26)	0.885	0.97 (0.83–1.15)	0.760
7	rs867186	A/G	0.831	0.06/0.06	0.87 (0.71–1.07)	0.202	1.01 (0.74–1.39)	0.942	0.81 (0.63–1.03)	0.087
8	rs9574	G/C	0.846	0.13/0.14	0.94 (0.82–1.08)	0.372	1.03 (0.83–1.28)	0.792	0.98 (0.83–1.15)	0.772
9	rs115542162	C/T	0.687	0.03/0.03	0.96 (0.73–1.25)	0.748	1.15 (0.77–1.74)	0.492	0.89 (0.65–1.22)	0.461

Aff, affected individuals; CI, confidence interval; HWE, Hardy-Weinberg equilibrium; MAF, minor allele frequency; OR, odds ratio; unaff, unaffected individuals.

a Results of logistic regression analyses assuming an additive mode of inheritance adjusted for gender, age, and ethnicity

**Figure 1:**
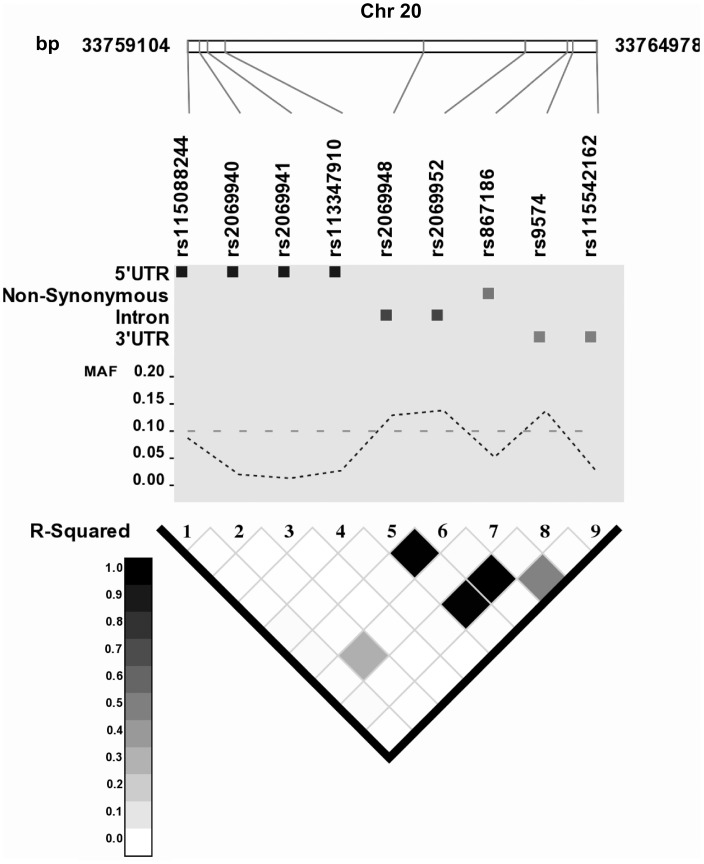
Linkage disequilibrium plot of the *PROCR* gene locus. The plot was generated on the basis of SNPs (MAF ≥1%) in healthy control individuals. Grey-scale squares represent pairwise r^2^ values ranging from 0–1.

Linkage disequilibrium (LD) between SNPs with MAFs ≥1% was found to be generally low, except for three pairs of SNPs, which were highly correlated (r^2^>0.99). These were two intronic variants, rs2069948 and rs2069952, and variant rs9574, which is located in the 3′-UTR. Other pairwise r^2^ values did not exceed 0.48 (Fig. 1).

Power calculations resulted in a power of 100%, 96%, and 100% to detect a genotype-phenotype association in the SM, CM, and SMA study groups, respectively, when assuming a genotype relative risk of at least 2 and a MAF ≥5%. In the case of low frequency SNPs (MAF ≥1%) the power obtained was 82% in the SM group, 19% in the CM group, and 66% in the SMA group.

Reconstruction of haplotypes generated seven full-length haplotypes with a global MAF ≥1%. Of these, haplotype PROCR-1 was the most prominent one with estimated frequencies of 68 and 69% in the control and case groups, respectively (Table 3). Haplotype PROCR-4 was found more frequently in cases than in controls (AF 4% in cases and 3% in controls). Whereas the difference was statistically significant in the haplotypic-specific score test (p = 0.04, p_empirical_ = 0.03), the global test-statistic produced a p-value of 0.20, indicating no association of full-length haplotypes with SM, CM, or SMA.

**Table 3:** Association tests of *PROCR* haplotypes with severe malaria, cerebral malaria, and severe malaria anemia.

			Severe malaria		Cerebral Malaria		Severe Malaria Anemia	
			N_cases_ = 1,905		N_cases_ = 431		N_cases_ = 1,226	
			N_controls_ = 1,866		N_controls_ = 1,866		N_controls_ = 1,866	
Haplotype	1-2-3-4-5-6-7-8-9^a^	Frequency (aff/unaff)	p-value^b^	Simulated^c^ p-value	p-value^b^	Simulated^c^ p-value	p-value^b^	Simulated^c^p-value
		N_cases_ = 1,905 N_controls_ = 1,866						
PROCR-1	GCCCTTAGC	0.69/0.68	0.296	0.294	0.407	0.405	0.326	0.291
PROCR-2	GCCCCCACC	0.14/0.14	0.357	0.348	0.820	0.832	0.690	0.676
PROCR-3	TCCCTTAGC	0.07/0.08	0.208	0.196	0.543	0.557	0.128	0.115
PROCR-4	GCCATTAGC	0.04/0.03	0.040	0.030	0.108	0.112	0.038	0.041
PROCR-5	GCCCTTGGT	0.03/0.03	0.925	0.929	0.504	0.502	0.589	0.608
PROCR-6	GGCCTTGGC	0.02/0.02	0.663	0.660	0.909	0.912	0.433	0.433
PROCR-7	GCTCTTAGC	0.01/0.01	0.697	0.710	0.265	0.293	0.784	0.808

Aff, affected individuals; unaff, unaffected individuals.

a Refers to SNP number as designated in Table 2.

b Results of haplotypic-specific score tests adjusted for gender, age, and ethnicity assuming an additive mode of inheritance.

c Simulation p-values are computed based on a permuted re-ordering of the trait and covariates in Haplo Stats [29].

The previously described functional *PROCR* haplotype A3, tagged by the G-allele of variant rs867186, was present in two haplotypes in the Ghanaian study group, PROCR-5 and PROCR-6, with frequencies of 3% and 2%, respectively. Neither the risk for SM nor for CM or SMA appeared to be influenced by any of the two haplotypes (Table 3). Haplotype PROCR-2, tagged by the C-allele of rs9574, which had previously been found to influence APC levels, had an estimated frequency of 14% in both, cases and controls. In our study group there was no sign for an association with risk of or protection from SM, CM, or SMA. This also applied to sub-haplotypes in the sliding-window analyses.

Four different algorithms were applied in order to test for an accumulation of rare variants in either the case or control groups. None of the approaches, including univariate and multivariate collapsing tests with varying thresholds, provided evidence for a joint effect of rare variants on the phenotypes tested (Table 4).

**Table 4:** PROCR rare variant analyses including SNPs with MAF ≤1%.

	Severe malaria	Cerebral malaria	Severe malaria anemia
	32 variants	27 variants	28 variants
	p-value	p-value	p-value
Univariate tests			
CMC	0.370	0.410	0.780
WSS	0.343	0.334	0.721
VT test	0.267	0.122	0.579
C-alpha	0.270	0.405	0.229
Multivariate tests a			
CMC	0.445	0.387	0.883
WSS	0.248	0.253	0.700
VT test	0.835	0.224	0.838

CMC, combined and multivariate collapsing; WSS, weighted sum statistic; VT, variable thresholds methods.

a Adjusted for age, gender, and ethnic group.

In conclusion, the discovered genetic variants in regulatory and coding regions of the *PROCR* gene were not found to influence susceptibility to SM, CM, or SMA in our study group.

## Discussion

After two recent reports on EPCR and its role in SM pathology, *PROCR*, the gene encoding EPCR, was considered a promising candidate to substantiate the *in-vitro* and *ex-vivo* data presented by a genetic association study. Here, we studied variation of *PROCR* in a large case-control group of SM from the Ashanti Region in Ghana, West Africa. None of the three approaches, including association testing of single SNPs, haplotypes, and rare variants, provided evidence for an association between variants in *PROCR* and the susceptibility to SM, CM, or SMA.

The lack of association may have several different explanations. First of all, it is conceivable, that, although against the current hypothesis, genetic variation in the gene does not alter susceptibility to SM phenotypes.

Second, not finding associations could be due to limitations of the study. For instance, additional genetic variation with functional relevance for *PROCR* gene expression, which may be located in *cis* or *trans* of the gene locus, could have remained undiscovered. As previous studies have shown *PROCR* gene transcription can be initiated at various sites. Sequences located −83 and −79 basepairs (bp) upstream of the translation initiation site have been described as alternative transcription start sites [6], [17]. Besides the common functional elements at the proximal promoter of *PROCR*, an additional regulatory element 5.5 kb upstream of the translation start was reported [18]. It was shown to exert enhancer activity in a cell-type specific manner. The possibility that genetic variation in this segment has an effect on *PROCR* gene expression and in turn on SM susceptibility appeared to be small because its sequence was found to be little diverse [18]. In this 500 bp enhancer region only one SNP (rs8119351) was found with a MAF>5% in genomic sequences of 669 individuals with African ancestry as part of the 1000 Genomes Project (http://www.1000genomes.org; assessed on 01^st^ August 2014). Nevertheless, when we tested this additional SNP in this study group no association was found (OR 0.80, 95% CI 0.61–1.04, p_corrected_ = 0.63).

Until now, there is one study that investigated variant rs867186 in the context of SM [19]. In that study, the authors reported evidence for an association of the GG genotype with protection from severe malaria in 707 Thai patients. However, results from that study are not totally convincing due to the fact that a statistically significant association was solely found when assuming a recessive mode of inheritance (MOI) (p = 0.026) and correction for multiple testing was disregarded. In our SM study group, when assuming the recessive MOI, the association test resulted in an OR of 1.32 (95% CI 0.39–4.47, p-value  = 0.658), clearly failing to show any genotype-phenotype association.

In addition to the association analysis described here we screened results from a genome-wide association study on SM which included 2,153 individuals from the same case-control study [20]. In that study approximately 800,000 SNPs per individual were genotyped throughout the genome. None of the genome-wide significant hits found was located in genes of molecules which have been described as part of the protein C anticoagulant and cytoprotective pathways [21]. Among others, these are protein C, Factor Va, Factor VIIIa, Thrombin, Thrombomodulin, and PAR-1.

The analyses of the potentially functional haplotypes, PROCR-2, -5, and -6, which were found to have frequencies of 14%, 3%, and 2%, respectively, did not show any evidence for association either. It is possible that these haplotypes do not exhibit the same function as described for Caucasians due to differing underlying regulatory mechanisms for *PROCR* gene expression in Africans. When comparing LD data and the haplotype substructure of the *PROCR* genomic region in Europeans with the Yoruba population from Nigeria, LD is considerably lower in the African subjects (1000 Genomes Project; www.ensembl.org), indicating inter-population genetic heterogeneity at this locus.

Further, a genotype-phenotype association in malaria may involve co-evolutionary effects between *P. falciparum* and its human host. Today there are numerous examples for highly specific host-pathogen interactions revealing the footprints of co-evolution at a molecular level [22], [23]. These include the invasion mechanisms of *P. falciparum* into the erythrocyte, which involves the RBC surface protein glycophorin C (GYPC). In the process of invasion, the *P. falciparum* erythrocyte-binding antigen 140 binds to GYPC on the surface of erythrocytes. A deletion in the gene of GYPC results in an RBC phenotype that cannot be invaded via this principal pathway [24]. This allele has reached a frequency of 46% in coastal areas of Papua New Guinea, where malaria is hyperendemic. Similarly, it is possible, that a specific structural variant of EPCR may be effective only against a certain type of PfEMP1 variants. In the case of a parasite strain expressing PfEMP1 conferring particularly strong binding to EPCR, a specific genetic host variant may be protective, and this variant would not necessarily be advantageous in infections with other parasites expressing other PfEMP1 variants. These highly specific protective mechanisms can only be detected when accounting for genetic and/or phenotypic substructure of the parasite population.

Another reason why existing genotype-phenotype associations may remain obscure is a lack of power. The power of a study depends on the number of study participants and MAFs and effect sizes of the alleles tested. Whereas the power was sufficient for SNPs in the SM study group, it was reduced to 19% when analysing alleles with frequencies <5% in the CM study group. Hence, the detection of associated low frequency variants (MAF <5%) with CM was underpowered, but for alleles with frequencies ≥5% the power was still appropriate with 96%.

The results presented here, although not providing evidence for associations between *PROCR* genetic variants and SM, CM, or SMA, do not preclude a role of *PROCR* genetic variation in malaria susceptibility in other settings. Moreover, the lack of association between genetic variation in *PROCR* and the phenotypes tested does not disagree with previous studies that support an important role of EPCR in SM and CM pathologies. Further studies, including gene expression studies of EPCR in African individuals would be helpful to determine expression quantitative trait loci (eQTLs) for EPCR and to find key regulatory mechanisms of its expression in individuals exposed to *P. falciparum* infection.

## Materials and Methods

### Severe malaria case-control group

The SM case-control group comprised 1,905 severe malaria patients and 1,866 healthy control individuals. The median age of cases and control individuals was 18 and 30 months, respectively, (ranges 1–156 months in the case group and 2–161 months in the control group). Of the severe cases, 431 (22.6%) presented with CM, 1,226 (64.4%) had SMA, 28.0% presented with hyperparasitemia, and 50.0% with prostration, with partly overlapping manifestations. Recruitment of study subjects, phenotyping, and DNA extraction has been described in detail elsewhere [16], [25].

### Variant discovery and genotyping

In order to screen the *PROCR* locus on chromosome 20 (chr20: 33,758,824–33,765,355) for genetic variants, DNA from 3,771 unrelated Ghanaian individuals (1,905 SM cases and 1,866 healthy controls) was used for HRM. Prior to the screen, genomic DNA had been whole-genome amplified by Genomiphi V2 DNA amplification kit (GE Healthcare). DNA samples were then amplified by PCR using primers that captured 1,100 bp upstream of the transcription start site, exons and their flanking regions, and 750 bp of the 3′-UTR. Oligonucleotides were designed using LightCycler Probe Design Software 2.0 (Roche Applied Science) against reference transcript NCBI NM_006404. Sequences of oligonucleotides and PCR conditions for HRM assays are listed in S1 Table. Previously unknown SNPs and singletons were confirmed by re-sequencing genomic DNA. In addition, 13 variants detected by HRM were genotyped by allele-specific hybridization in a Roche LightCycler device (S2 Table).

### Association analyses of SNPs with MAFs ≥1%

Logistic regression was used to test for association of nine variants in the case-control study assuming an additive MOI in PLINK v1.07 [26]. Ethnic group, age, and gender were used as covariates in the regression model. Logistic regression analyses did not account for HbS or HbC allele carrier status of individuals. In order to account for multiple testing we used a correction factor of 7, hence, a p-value <0.007 (0.05/7) was considered significant. A factor of seven was applied because three of the nine SNPs tested were highly correlated to each other (pairwise r^2^>0.99; see results section), thereby reducing the number of independent statistical comparisons to seven. Variants were tested for fulfilling the Hardy-Weinberg equilibrium (HWE) in PLINK. Power for detecting genetic effects of variants in the case-control study was estimated with CATS [27]. For power estimations, a disease prevalence of 2% for severe malaria and p-value <0.007 were assumed.

### Calculation of LD

LD-Plus was used to generate the LD plot of the *PROCR* genomic region [28]. The LD calculation was based on the control individuals and included *PROCR* variants with MAF ≥1%.

### Haplotype-based association testing

Full-length haplotype analyses were done for the case-control group with the Haplo Stats package v1.4.4 [29] in R (version 3.1.0; http://www.r-project.org), including reconstructed haplotypes with estimated global frequencies ≥1%. In addition, sub-haplotypes were evaluated in sliding-window analyses capturing a minimum of two and a maximum of eight alleles. SM, CM, and SMA were used as phenotypes, and the three covariates ethnic group, age, and gender were included in the model. Empirical p-values were computed using default values.

### Association analyses of rare variants (MAF <1%)

We tested for association between rare variant carrier status at *PROCR* and SM, CM, or SMA in the study group. Four methods were used to assess the overall genetic burden due to rare variants, namely (i) the combined and multivariate collapsing (CMC) method described by Li and Leal [30], (ii) the weighted sum statistic (WSS) described by Madsen and Browning [31], (iii) the variable-threshold (VT) model described by Price *et al*. [32], and (iv) the C(α) test described by Neale *et al*. [33], all integrated in *variant tools* [34]. For these analyses variants were pooled on the basis of their MAF and their cumulative effect was tested in both univariate and multivariate analyses, in which age, gender, and ethnic group were included as covariates.

### Ethics statement

Ethical clearance was granted by the Committee for Research, Publications and Ethics of the School of Medical Sciences, Kwame Nkrumah University of Science and Technology, Kumasi, Ghana. All procedures were explained to parents or guardians of the participating children in the local language, and written or thumb-printed informed consent was obtained.

## Supporting Information

S1 Table **Oligonucleotides and PCR conditions for high resolution melting assays.**
(DOC)

S2 Table **Oligonucleotides and PCR conditions for SNP genotyping.**
(DOC)
