# Supplementary material for: Endothelial Protein C Receptor Gene Variants Not Associated with Severe Malaria in Ghanaian Children
Source: PLoS One. 2014 Dec 26;9(12):e115770. doi: 10.1371/journal.pone.0115770 (PMC4277309; doi:10.1371/journal.pone.0115770)
Supplement: S1 Table — Oligonucleotides and PCR conditions for high resolution melting assays. (DOC) [file pone.0115770.s001.doc]

Table S1. Oligonucleotides and PCR conditions for high resolution melting assays

|  | Generegion | Length of Amplicon  [bp] | Oligonucleotides | Annealing temperature | Additional or deviant reagents in 10 μl reaction |
| --- | --- | --- | --- | --- | --- |
| 1 | Promoter Part 1 | 251 | | PROCR_Prom1-F CAGAATCTTGCCGCATGACCCAGG | | --- | | PROCR_Prom1-R CATGCTGCTGGCACTTTGGGAGG | | HotStart  65°C | 2.5 mM MgCl_2_  5% DMSO  5x Qiagen Q-Solution  0.5 µM Forward  0.5 µM Reverse |
| 2 | Promoter Part 2 | 308 | | PROCR_Prom2-F GCCTCCCAAAGTGCCAGCAG | | --- | | PROCR_Prom2-R AAGCAATCTTTTTGAAACTGAGGTCA | | HotStart  60°C | 2 mM MgCl_2_  5% DMSO  5x Qiagen Q-Solution  0.5 µM Forward  0.5 µM Reverse |
| 3 | Promoter Part 3 | 218 | | PROCR_Prom3_F2 CGCTGAAATTTTGTATTCTGTCCTAT | | --- | | PROCR_Prom3_R2 AGTCATAAAATGAGTGTAAAGCAGTTC | | 58°C | 2.5 mM MgCl_2_  5 µg BSA |
| 4 | Promoter Part 4 | 213 | | PROCR_Prom4_F2 ACAGATTGCTCATAATTCTCTCCTATT | | --- | | PROCR_Prom4_R2 CAGGGACTTGCCCAAAGTTTATATATC | | 58°C | 3 mM MgCl_2_  5 µg BSA  1x Solis Solution S |
| 5 | Promoter Part 5 | 217 | | PROCR_Prom5-F TGGTAAAATGTAGCCATTATACTGG | | --- | | PROCR_Prom5-R GAGAAGGGCTTGGTGAGAAG | | HotStart  55°C | 2.5 mM MgCl_2_  5 µg BSA  1x Solis Solution S |
| 6a | Promoter Part 6  Outer PCR | 535 | PROCR_Prom5-F TGGTAAAATGTAGCCATTATACTGG  PROCR_ex1_1-R GAAGTTGAGGCTCCGGAC | HotStart 55°C 20 cycl., subsequent digestion with SAP + Exonuclease I | 5µl reaction  2.0 mM MgCl_2_  5% DMSO |
| 6b | Promoter Part 6  Nested PCR | 235 | | PROCR_Prom6-F GGAGTGTGCTCTAAGTTGAAAGTAG | | --- | | PROCR_Prom6-R AGTGTTATTTCTACTGTGTCTCATTTCC | | HotStart  53°C | 2.5 mM MgCl_2_  3% DMSO  0.4 µM Forward  0.4 µM Reverse |
| 7 | Exon 1 Part 1 | 195 | | PROCR_ex1_1-F CTAGTAGGAAATGAGACACAGTAGAAA | | --- | | PROCR_ex1_1-R GAAGTTGAGGCTCCGGAC | | HotStart  58°C | 2.0 mM MgCl_2_  5% DMSO  1x Qiagen Q-Solution  0.5 µM Forward  0.5 µM Reverse |
| 8 | Exon 1 Part 2 | 219 | | PROCR_ex1_2-F CGGTCCTCACTTCTCTTTTCC | | --- | | PROCR_ex1_2-R CAGATAGACTGAGATTCTCCAGAAC | | HotStart  58°C | 2.5 mM MgCl_2_  5% DMSO  1x Qiagen Q-Solution  0.5 µM Forward  0.5 µM Reverse |
| 9 | Exon 2 Part 1 | 228 | | PROCR_ex2_1-F_vs3 GCCAGCCTCGAGGTAGGGGGTTAT | | --- | | PROCR_ex2_1-R_vs3 GCAGCTGAATGATCGTGGTGTTGGT | | HotStart-Touchdown  11 cycl.  66-56°C+  45 cycl., 55 °C | 3 mM MgCl_2_  5% DMSO  1 µM Forward  1 µM Reverse |
| 10a | Exon 2 Part 2 Outer | 628 | | PROCR_ex2_out-F CTGTCCTGTCCTCCTGGCAGAGTT | | --- | | PROCR_ex2_out-R TCTTAGCGGGGACAACTGCCTCTC | | HotStart  55°C,20 cycl.  subsequent digestion with SAP + Exonuclease I | 5µl reaction  2.0 mM MgCl_2_  5% DMSO |
| 10b | Exon 2 Part 2  Nested | 252 | | PROCR_ex2_2-F_vs3 CCTATCACGTGTGGTACCAGGGCA | | --- | | PROCR_ex2_2-R_vs3 CCACTAGCCCCGCCCAGACC | | HotStart  55°C | 4µl reaction  2.5 mM MgCl_2_  3% DMSO  0.4 µM Forward  0.4 µM Reverse |
| 11 | Exon 3 Part 1 | 266 | | PROCR_ex3_1-F ACTCTTGCCTTCTCATGTTCTTTTC | | --- | | PROCR_ex3_1-R GTGAAGGTGACCACTCCGG | | HotStart  58°C | 2.5 mM MgCl_2_  5% DMSO  0.5 µM Forward  0.5 µM Reverse |
| 12 | Exon 3 Part 2 | 294 | | PROCR_ex3_2-F TTCTTCGAAGTGGCTGTGAATG | | --- | | PROCR_ex3_2-R CATCCATCCTTCAGGTCCATC | | HotStart  58°C | 2.5 mM MgCl_2_  5% DMSO  0.5 µM Forward  0.5 µM Reverse |
| 13 | Exon 4 Part 1 | 257 | | PROCR_ex4_1-F CAGAAACGCTTTGGGGTTTG | | --- | | PROCR_ex4_1-R TGAAACTTTCCCTTGCCAGC | | 58°C | 2.5 mM MgCl_2_  1x Solis Solution S  5 µg BSA |
| 14 | Exon 4 Part 2 | 277 | | PROCR_ex4_2-F TGTAGGCATCTTCCTGTGCAC | | --- | | PROCR_ex4_2-R AGACTAATTCAGCAAAGCATACACG | | HotStart  58°C | 3.0 mM MgCl_2_  1x Solis Solution S  5% DMSO |
| 15 | Exon 4 Part 3 | 260 | | PROCR_ex4_3-F ACATCTGCCCACTGAAGATTTG | | --- | | PROCR_ex4_3-R AATGTTGTATTTTGATTGTGGATGACT | | 58°C | 3.0 mM MgCl_2_  1x Solis Solution S  5 µg BSA |
| 16 | Exon 4 Part 4 | 218 | | PROCR_ex4_4-F TTGGGGCAGGAAGCCTATG | | --- | | PROCR_ex4_4-R GGAGGAGATAACAATGCCTTAAATATAAG | | 58°C | 2.5 mM MgCl_2_  1x Solis Solution S  5 µg BSA |
| 17 | Exon 4 Part 5 | 182 | | PROCR_ex4_5-F_vs2 TAACACGAAGAAGTGGTGGAAATG | | --- | | PROCR_ex4_5-R_vs2 AAAGAATGAGTGAGAAATAAGGAAGCAG | | HotStart-Touchdown  11 cycl.  65-56°C  +45 cycl.  55 °C | 2.5 mM MgCl_2_  5 µg BSA  1x Qiagen Q-Solution  0.5 µM Forward  0.5 µM Reverse |
| 18 | 3'UTR | 345 | | PROCR_3'UTR-F CTGCTTCCTTATTTCTCACTCATTCTTT | | --- | | PROCR_3'UTR-R GTCAGATTGCACTGGGTCTAAGAAT | | HotStart  60°C | 1.5 mM MgCl_2_  5% DMSO  0.5 µM Forward  0.5 µM Reverse |

Unless otherwise specified the following reagents are used in all reactions (PCR components: Solis Biodyne): 1x PCR-Buffer, 200 µM dNTPs, 1 unit Taq, 0,3 µM of each primer (Biomers), 1x EvaGreen, 20 ng DNA. Cycle protocol: 3 min initial denaturation 94°C (for HotStart reactions 15 min); 45 cycl. 30 sec denaturation 94°C, 1 min annealing as mentioned below, 1 min 72°C (for HotStart reactions 68°C), 5 min final extension 72°C (for HotStart reactions 68°C)
