# Supplementary material for: Endothelial Protein C Receptor Gene Variants Not Associated with Severe Malaria in Ghanaian Children
Source: PLoS One. 2014 Dec 26;9(12):e115770. doi: 10.1371/journal.pone.0115770 (PMC4277309; doi:10.1371/journal.pone.0115770)
Supplement: S2 Table — Oligonucleotides and PCR conditions for SNP genotyping. (DOC) [file pone.0115770.s002.doc]

Table S2. Oligonucleotides and PCR conditions for SNP genotyping

|  | rs_ID | Oligonucleotides | Annealing temperature | Additional or deviant reagents in 10 μl reaction |
| --- | --- | --- | --- | --- |
| 1 | rs112681065 | PRO_356-F CATGCTCGGAGGAGTGACT  PRO_356-R GAAATAGGACAGAATACAAAATTTCAGCG  PRO_356-S TATCCAAAACTCATCATAGAAACATACA-6FAM  PRO_356-A BMN5-CACAAAACCAAAGCACAMATATACAAC-Spacer C3 | 55°C | 1.5 mM MgCl_2_  5 µg BSA  1x Solis Solution S |
| 2 | rs115088244 | | PRO_405-F TTCCTAGAGTAAGGGACGCATTTTAC | | --- | | PRO_405-R ACTGAGGTCATAATTTATTAGTGGACAATG | | PRO_405-S ATATACAACTGAGCAAATATTTCA-6FAM | | PRO_405-A BMN5-ACATAACASTTTCTCTTACTAAGGGTGAC-Spacer C3 | | 55°C | 2.0 mM MgCl_2_  5 µg BSA  1x Solis Solution S |
| 3 | rs2069940 | | PRO_573-F TTTCTCTTACTAAGGGTGACGCG | | --- | | PRO_573-R AGTTCACTGCAGCATGTTTTT | | PRO_573-S BMN5-AGGTAACCAATCATCTTCTGAG-Spacer C3 | | PRO_573-A ATAAATTATGACCTCAGTTTCAAAAAGATTGCT-6FAM | | 58°C | 1.5 mM MgCl_2_  5 µg BSA  1x Solis Solution S |
| 4 | rs2069941 | | PRO_687-F CTTCTGAGATTTATACAGATTGCTC | | --- | | PRO_687-R AGGGACTTGCCCAAAGTTTATATATC | | PRO_687-S CCAAGATCCCGGATTATGT-6FAM | | PRO_687-A BMN5-TTGTTATTTACTTAAAATTCTGGTAAAATGTAGCCAT-Spacer C3 | | 58°C | 2.0 mM MgCl_2_  5 µg BSA |
| 5 | rs113347910 | | PRO_940-F TGGTAAAATGTAGCCATTATACTGG | | --- | | PRO_940-R GAAGTTGAGGCTCCGGAC | | PRO_940-S GCCCTTCTCCCCCTTTTC-6FAM | | PRO_940-A BMN5-CTCCCTGTTCCTGGTTCCTAGGAAG-Spacer C3 | | HotStart  55°C | 2.5 mM MgCl_2_  5 µg BSA  1x Solis Solution S  0.2 µM Forward  1 µM Reverse |
| 6 | rs2069948 | | PRO_3790-F GCCAGCCTCGAGGTAGGGGGTTAT | | --- | | PRO_3790-R GCAGCTGAATGATCGTGGTGTTGGT | | PRO_3790-S CCCAGGCTGAAGCTGACT-6FAM | | PRO_3790-A BMN5-GCCCGCAGGCCTCCAAAGAC-Spacer C3 | | HotStart-Touchdown  11 cycl.  66-56°C+  45 cycl.  55 °C | 3.0 mM MgCl_2_  5% DMSO  0.25 µM Forward  1 µM Reverse |
| 7 | rs2069952 | | PRO_5252-F ACTCTTGCCTTCTCATGTTCTTTTC | | --- | | PRO_5252-R GTGAAGGTGACCACTCCGG | | PRO_5252-S CACAGTCCCCTGACCCTGA-6FAM | | PRO_5252-A BMN5-GTCTATCCACAGTTCCTCTGACCATCC-Spacer C3 | | 55°C | 1.5 mM MgCl_2_  5 µg BSA  1x Solis Solution S |
| 8 | rs867186 | | PRO_5855-F CAGAAACGCTTTGGGGTTTG | | --- | | PRO_5855-R TGAAACTTTCCCTTGCCAGC | | PRO_5855-S BMN5-ATGAAACCGCCCACCAGG-Spacer C3 | | PRO_5855-A ATGCCTACAGCCACACCAGCAA-6FAM | | 55°C | 1.5 mM MgCl_2_  5 µg BSA  1x Solis Solution S |
| 9 | rs9574 | | PRO_5933-F TGTAGGCATCTTCCTGTGCAC | | --- | | PRO_5933-R AGACTAATTCAGCAAAGCATACACG | | PRO_5933-S BMN5-TCTGAGGGGGCTGGAG-Spacer C3 | | PRO_5933-A CCAGCCTCCATCAATCCAGCCC-6FAM | | HotStart-Touchdown  11 cycl.  60-50°C  20/30/30 sec;+  45 cycl.  53 °C  20/30/30 sec | 2.0 mM MgCl_2_  5 µg BSA  1x Solis Solution S  1 µM Forward  0.2 µM Reverse |
| 10 | rs115542162 | | PRO_6279-F TTGGGGCAGGAAGCCTATG | | --- | | PRO_6279-R GGAGGAGATAACAATGCCTTAAATATAAG | | PRO_6279-S ACCAAATAAACAAGTCATCCAC-6FAM | | PRO_6279-A BMN5-TCAAAATACAACATTCAATACTTCCAGGTGTG-Spacer C3 | | 55°C | 1.5 mM MgCl_2_  5 µg BSA  1x Solis Solution S |
| 11a | rs141487483  Outer-PCR | | PROCR_ex2_out-F CTGTCCTGTCCTCCTGGCAGAGTT | | --- | | PROCR_ex2_out-R TCTTAGCGGGGACAACTGCCTCTC | | HotStart 55°C  20 cycl. digestion with SAP + Exonuclease | 0,25u QiagenHotStarTaq  5µl reaction 2.0 mM MgCl2  5% DMSO  0.3 µM Forward+Reverse |
| 11b | rs141487483  Nested-PCR | | PRO_3965-F CCTATCACGTGTGGTACCAGGGCA | | --- | | PRO_3965-R CCACTAGCCCCGCCCAGACC  PRO_3965-S BMN5-AGCTCCCGGGCTCC -Spacer C3  PRO_3965-A CCACTCTGCGTGCGCGC-6FAM | | HotStart  55°C | 0,25u QiagenHotStarTaq  Outer-PCR as template  2.0 mM MgCl2  3% DMSO  0.5 µM Forward  0.13 µM Reverse  0.3 µM Probes |
| 12 | rs148966205 | | PRO_5759-F CAGCTTCAGTCAGTTGGTAAACGG | | --- | | PRO_5759-R GGGGCTGGAGAGTAATTAACATCG | | PRO_5759-S ATGGACTCCATGGGGGC-6FAM | | PRO_5759-A BMN5- ATTCTTCGGGCTAACTCTTTGCATGTTCT- Spacer C3 | | 58°C | 1.5 mM MgCl2  5 µg BSA  1x Solis Solution S |
| 13 | rs1485801152 | | PRO_5843-F CAGCTTCAGTCAGTTGGTAAACGG | | --- | | PRO_5843-R GGGGCTGGAGAGTAATTAACATCG | | PRO_5843-S BMN5- CCAGGACGCCCAGGACC -Spacer C3 | | PRO_5843-A CCACACCAGCAATGATGAAACNGCC-6FAM | | 58°C | 2.0 mM MgCl2  5 µg BSA  1x Solis Solution S |

Unless otherwise specified the following reagents are used in all reactions (PCR components: Solis Biodyne): 1x PCR-Buffer, 200 µM dNTPs, 1 unit Taq, 0. 2 µM of each dye labeled probe (Biomers), 0.13 and 0.5 µM Forward and Reverse Primer (Biomers) or vice versa, depending on orientation of the probes, 20 ng DNA

Cycle protocol: 3 min initial denaturation 94°C (for HotStart reactions 15 min); 45 cycl. 30 sec denaturation 94°C, 1 min annealing as mentioned below, 1 min 72°C (for HotStart reactions 68°C), 5 min final extension 72°C (for HotStart reactions 68°C)
